# External root resorption with the self-ligating Damon system—a retrospective study

**DOI:** 10.1186/s40510-016-0133-1

**Published:** 2016-07-01

**Authors:** Roberta Heiffig Handem, Guilherme Janson, Murilo Matias, Karina Maria Salvatore de Freitas, Darwin Vaz de Lima, Daniela Gamba Garib, Marcos Roberto de Freitas

**Affiliations:** Department of Stomatology and Radiology, Bauru Dental School, University of São Paulo, Bauru, Brazil; Department of Orthodontics, Bauru Dental School, University of São Paulo, Alameda Octávio Pinheiro Brisolla 9-75, Bauru, SP 17012-901 Brazil; Department of Orthodontics, Ingá Dental School, Maringá, Brazil; Private practice, Cuiabá, Brazil

**Keywords:** Root resorption, Periapical radiograph, Self-ligating, Orthodontic

## Abstract

**Background:**

The aim of this study was to compare the degree of external apical root resorption (EARR) in patients treated with self-ligating Damon appliances and with conventional preadjusted appliances.

**Methods:**

The sample comprised 52 patients, divided into two groups. Group 1 consisted of 25 patients treated with self-ligating Damon appliances, with an initial age of 16.04 years, final age of 18.06 years, and treatment time of 2.02 years. Group 2 consisted of 27 patients, treated with conventional preadjusted appliances, with an initial age of 16.77 years, final age of 18.47 years and treatment time of 1.70 years. The groups were matched regarding the initial and final ages, treatment time, type of malocclusion, and treatment protocol without extractions. Root resorption was evaluated on periapical radiographs of the maxillary and mandibular incisors at the end of orthodontic treatment with the scores of Levander and Malmgren. Intergroup comparisons of root resorption were performed with Mann-Whitney tests.

**Results:**

No significant difference in the degree of root resorption between the two groups was found.

**Conclusions:**

Similar degrees of resorption can be expected after non-extraction treatment with Damon self-ligating or conventional preadjusted appliances.

## Background

External apical root resorption (EARR) is often thought of as an iatrogenic consequence of orthodontic treatment [1–4]. In fact, a faster correction of the malocclusion could lead to undesirable side effects, such as root resorption, which is a great concern for orthodontists [5]. Several factors have been investigated, and intrusion and retraction have been considered the main causes of EARR [4, 6–11]. According to this and the fact that mechanical forces are a key factor in the occurrence of EARR, studies have shown that the appliance or technique used for an orthodontic treatment can be related to the degree of EARR [11–14]. On the other hand, studies have demonstrated that, in general, light forces usually tend to cause less resorption [15–17].

In the last decade, there has been a significant increase in the number of self-ligating appliance systems available for orthodontists. The Damon system (Ormco, Glendora, CA) is based on the use of a passive self-ligating bracket and superelastic nickel-titanium wires [18]. This system is attractive due to the promise of excellent treatment of almost every patient, providing treatment without extractions, orthognathic surgery, palatal expansion, and pain, within a short period of time [19]. The Damon system presents important advances in terms of strength and usability. It is especially emphasized that this system, with low friction brackets, applies only light forces to move the teeth [20]. It has been demonstrated that during the initial leveling and alignment stage, root resorption with this system is similar to conventional preadjusted edgewise bracket systems [21, 22]. However, root resorption after complete orthodontic treatment with the Damon system has not been investigated. Perhaps, the root resorption similarity at the leveling and alignment stage may have been consequent to the light wires initially used in both systems and to the short period of time of evaluation [12, 15–17, 20, 23, 24].

Therefore, the purpose of this retrospective study was to compare the degree of EARR in patients treated with the Damon self-ligating system with patients treated with conventional brackets, after complete orthodontic treatment.

## Methods

### Material

This study was approved by the Ethics in Research Committee of the University of São Paulo, Bauru Dental School, Brazil.

The sample size was calculated based on an alpha significance level of 0.05 and a beta of 0.2 to achieve 80 % of power to detect a mean difference of 0.32 as with standard deviation of 0.39 in the final score of root resorption [25]. The sample size calculation showed that 24 patients were needed, and to increase the power even more, it was decided to select 25 and 27 patients for each of the experimental groups.

Fifty-two patients were used in this retrospective study, regardless of race and sex. Only class I malocclusion patients, with mild to moderate crowding, and with all permanent teeth erupted, up to the first molars, who were treated non-extraction were included in the sample. Patients that presented apical root resorption or endodontic treatment at the pretreatment stage were excluded, as well as patients whose orthodontic records were incomplete. Poor quality radiographs were also eliminated. None of the patients were re-treatment cases.

Group 1 consisted of 25 patients (13 male; 12 female), with an initial mean age of 16.04 years and final mean age of 18.06 years, treated with the 0.022 × 0.028-in. preadjusted 3MX Damon System™ self-ligating brackets, (Ormco, Glendora, CA). Nine patients were treated at the orthodontic clinic of the University of São Paulo, Bauru Dental School, Brazil and, 16 were treated in a private clinic. The mean treatment time was 2.02 years. Non-extraction treatment with the Damon system is characterized by beginning leveling and alignment with round 0.014 or 0.016 in., followed by rectangular 0.016 × 0.025, 0.018 × 0.025, and 0.019 × 0.025 in. Nitinol thermo-activated archwires. Subsequently, rectangular 0.017 × 0.025 or 0.019 × 0.025 in. stainless steel archwires are used. The wire sequence is dependent upon the needs of each patient. Deep overbites are usually corrected by reversing and accentuating the curve of Spee of the stainless steel archwires since the beginning, until an overcorrection is obtained, when necessary. This overcorrection is maintained by accentuating and reversing the curve of Spee in the rectangular archwire as well. After leveling and alignment, the finishing procedures were performed.

Group 2 consisted of 27 patients (13 female; 14 male), with an initial mean age of 16.77 years and final mean age of 18.47 years, treated with 0.022 × 0.028 in. preadjusted Roth prescription, non-self-ligating appliances, at the University of São Paulo, Bauru Dental School, Brazil. The mean treatment time was 1.70 years. Non-extraction treatment with the preadjusted appliances is characterized by leveling and alignment with round 0.012, 0.014, and 0.016 in. NiTi archwires. Subsequently, round 0.016, 0.018, and 0.020 in. stainless steel archwires are used, followed by rectangular 0.019 × 0.025 or 0.021 × 0.025 in. stainless steel archwires. The archwire sequence is dependent upon the treatment needs. Deep overbites are usually corrected by reversing and accentuating the curve of Spee of the stainless steel archwires since the beginning, until an overcorrection is obtained. This overcorrection is maintained by accentuating and reversing the curve of Spee in the rectangular wire as well. Thereafter, finishing procedures were conducted.

### Methods

**Fig. 1:**
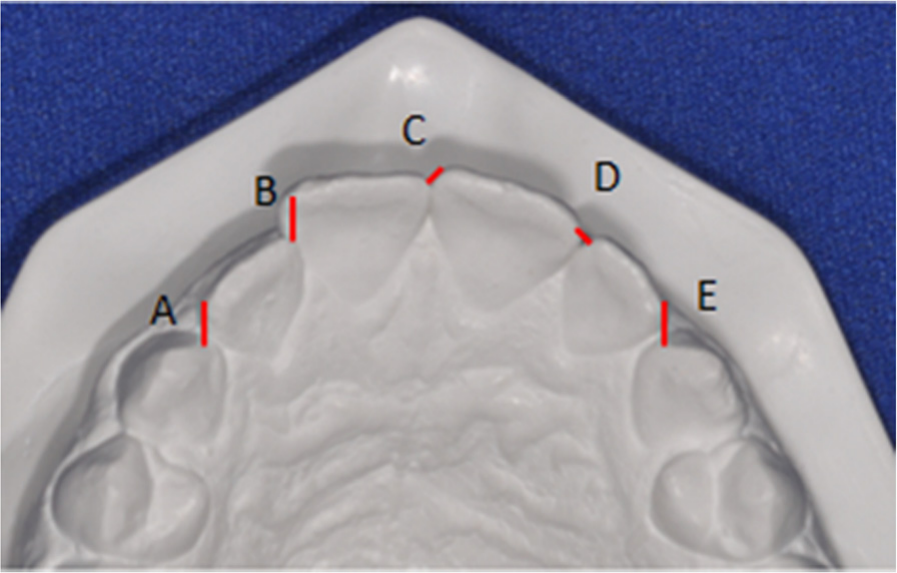
Modified Little’s irregularity index for the maxillary arch/*A* + *B* + *C* + *D* + *E*

Because both groups could present mild to moderate crowding at the pretreatment stage, the mandibular and maxillary crowding were measured according to Little’s irregularity index [26] and later compared between the groups to ascertain that they were comparable regarding these variables. This index corresponds to the sum, in millimeters, of the five distances between the anatomic contact points from the mesial of the mandibular right canine through the mesial of the left canine. The index was similarly measured in the maxillary arch [27] (Fig. 1).

To quantify resorption, pre- and posttreatment periapical radiographs of the maxillary and mandibular incisors, totaling 208 radiographs, were examined. The decision to work only with the incisors was because they have a higher prevalence of resorption [28–32].

The periapical radiographs of the patients treated at the University were obtained by a single operator with the DABI 70 Spectro 1070X X-ray machine, set up for 70 kV, 10 mA, and an exposure time of 1 s, with the long cone-paralleling technique. Kodak Ektaspeed EP 21 films were used, and the angles were obtained by an intraoral XCP positioner (Rinn-Dentisply). The radiographs of the private practice patients were obtained by different operators, with a Kavo Express X-ray machine, set up for 65 kV, 7 mA, and an exposure time of 1 s with the same type of positioner mentioned above.

**Fig. 2:**
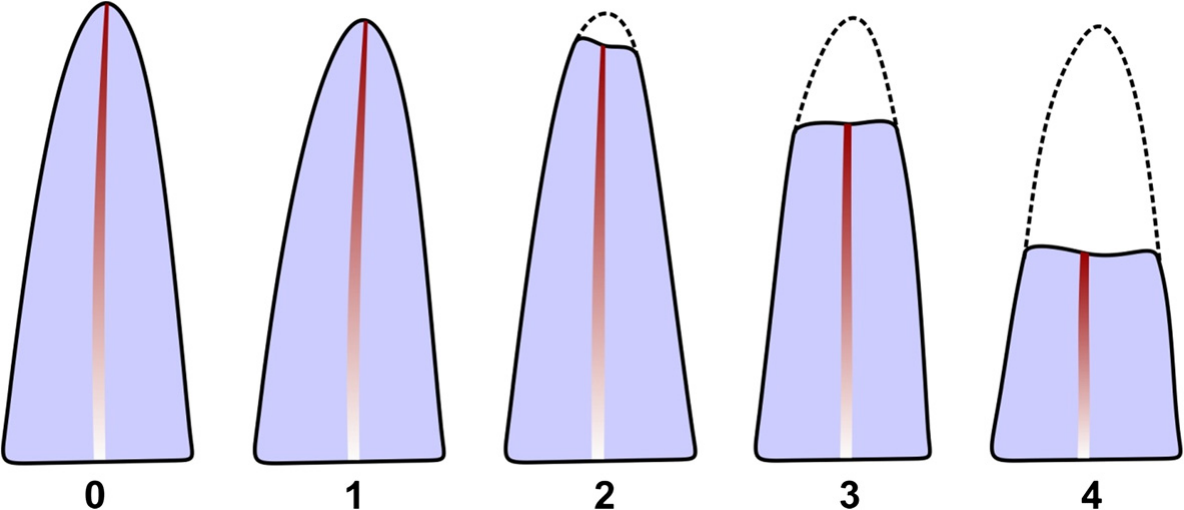
Ranking levels of the external apical root resorption by Levander and Malmgren [10]. Grade *0*: absence of root resorption; grade *1*: mild resorption, root with its normal length, and only an irregular contour; grade *2*: moderate resorption, small area of root loss with the apex exhibiting an almost straight contour; grade *3*: accentuated resorption, loss of almost one third of root length; and grade *4*: extreme resorption, loss of more than one third of the root length

Standardization of the radiographs taken by different operators was not a concern since resorption was evaluated by the score system of Levander and Malmgren [10] that classifies it in five grades (Fig. 2).

The periapical radiographs were scanned with a 35-mm slide scanner (Polaroid Sprint Scan 35 Plus 3), with 675 dpi of resolution in a 1:1 (100 %) scale. Later, the images were standardized and analyzed in Adobe Photoshop CS6. The images were allowed to be magnified up to 300 % without quality loss. Measurements were recorded in “TIFF” format. The radiographic analysis was blindly performed by one examiner in a dark room. The resorption grade of each tooth was recorded on each patient’s chart. Other information was later recorded on the chart such as age, sex, and technique used.

The other records were used to determine patient’s sex and age at the pretreatment stage, the type of treatment undertaken, and the orthodontic technique that was used.

### Error study

Fifteen patients were randomly selected and the mandibular and maxillary irregularity indexes were remeasured and their teeth were scored again regarding the resorption level, by the same examiner, after 30 days. For the irregularity indexes, random errors were calculated according to Dahlberg’s formula (Se^2^ = Σd^2^/2n), where Se^2^ is the error variance and *d* is the difference between two determinations of the same variable [33]. Systematic errors were evaluated with dependent *t* tests, at *P* < 0.05 [34]. The repeated measurements of root resorption were tested by Kappa coefficient of agreement.

### Statistical analyses

Normal distribution of the variables was evaluated with Kolmogorov-Smirnov tests. The results showed normal distribution for all variables.

To evaluate intergroup comparability regarding sex distribution, initial and final ages, treatment time and Little’s irregularity index, chi-square, and *t* tests were used, respectively.

To compare the intergroup resorption scores, Mann-Whitney tests were used. The percentage of teeth with the several resorption scores was calculated for each group and for the groups combined. All statistical analyses were performed with Statistica software (Statistica for Windows – Release 7.0 - Copyright Statsoft, Inc. Tulsa, OK). Results were considered statistically significant at *P* < 0.05.

## Results

There were no significant systematic errors and the random errors were 0.08 and 0.36 for the maxillary and mandibular irregularity indexes. Kappa coefficient regarding resorption evaluation was 0.749, showing substantial agreement.

The groups were comparable regarding sex distribution, initial and final ages, treatment time, and maxillary and mandibular irregularity indexes (Table 1).

**Table 1:** Intergroup comparability (chi-square and *t* tests)

	Male		Female		Total
Damon	13		12		25
Conventional	15		12		27
Total	28		24		52
	X^2^ = 0.06 DF = 1 *P* = 0.797^a^				
	Group 1—Damon *n* = 25		Group 2—conventional *n* = 27		
	Mean	SD	Mean	SD	*P*
Initial age	16.04	5.20	16.77	5.32	0.623^b^
Final age	18.06	5.51	18.47	5.43	0.788^b^
T. time	2.02	0.68	1.70	0.56	0.078^b^
MxII.	6.60	3.68	5.13	1.66	0.065^b^
MdII.	6.36	2.93	5.40	1.60	0.144^b^

*MxII.* maxillary irregularity index, *MdII.* mandibular irregularity index

^a^Chi-square test

^b^ *t* test

There was no intergroup difference regarding the amount of root resorption at the end of treatment (Table 2).

**Table 2:** Intergroup comparison of the amount of root resorption at the end of treatment (Mann-Whitney tests)

Variable	Group 1—Damon *N* = 25		Group 2—conventional *N* = 27		*P*
	Mean (median)	Interquartile deviation	Mean (median)	Interquartile deviation	
EARR 12	0.72 (0.00)	1.00	0.70 (1.00)	1.00	0.978
EARR 11	0.72 (0.00)	1.00	0.59 (0.00)	1.00	0.653
EARR 21	0.88 (1.00)	1.00	0.66 (0.00)	1.00	0.480
EARR 22	0.80 (1.00)	1.00	0.74 (1.00)	1.00	0.595
EARR 32	0.56 (0.00)	1.00	0.55 (0.00)	1.00	0.833
EARR 31	0.60 (1.00)	1.00	0.62 (1.00)	1.00	1.000
EARR 41	0.64 (1.00)	1.00	0.66 (0.00)	1.00	0.869
EARR 42	0.48 (0.00)	1.00	0.40 (0.00)	1.00	0.673

In group 1 (Damon), of the 200 evaluated teeth, 93 showed no radiographically visible root resorption, 83 showed slight resorption, 20 moderate resorption, four severe resorption, and none showed extreme resorption. In group 2 (non-self-ligating), of the 216 evaluated teeth, 114 showed no radiographically visible root resorption, 74 showed slight resorption, 24 moderate resorption, four severe resorption, and none showed extreme resorption (Table 3).

**Table 3:** Resorption scores found in groups 1 and 2

Groups	Scores									
	0		1		2		3		4	
	*N*	%	*N*	%	*N*	%	*N*	%	*N*	%
Group 1	93	46.5	83	41.5	20	10	4	2	0	0
Group 2	114	52.7	74	34.2	24	11.1	4	1.85	0	0

*N* number of teeth

From the total sample, 416 teeth were analyzed and 207 (49.7 %) had no root involvement (level 0), 157 (37.7 %) showed mild resorption (level 1), 44 (10.5 %) had moderate resorption, 8 (1.92 %) showed accentuated resorption (level 3) and no teeth had extreme resorption (level 4, Table 4).

**Table 4:** Number and percentage of all evaluated teeth with the different levels of external apical root resorption

Resorption	*n*	%
No root involvement	207	49.7
Mild	157	37.7
Moderate	44	10.5
Accentuated	8	1.9

## Discussion

Patients of groups 1 and 2 were treated by different professionals. It is difficult to find a significant sample treated and concluded by a single professional, with the same technique, especially if it has been recently developed. However, this should not interfere in the comparison because studies have shown that this does not influence the results [30, 35–38].

Root resorption was evaluated with periapical radiographs, which is the method used by most authors. Periapical radiographs provide more details and better image definition than panoramic radiographs and expose the patient to less radiation [8, 9, 39–41]. The choice to use the incisors was because they are usually more affected by resorption and they are uniradicular [31, 32, 42, 43].

The evaluation method used in this research, proposed by Levander and Malmgren [10] ranks resorption in scores. That does not depend on the standardization of the initial radiograph and thus is evaluated by the magnitude, which becomes its main advantage. Another relevant factor is that the intraobserver errors showed substantial agreement level, thus reducing the possibility of error.

The amount of root resorption was similar in the groups, contrary to statements that the Damon self-ligating system would produce less root resorption (Table 2). These statements were based on the fact that with low-friction appliances, the necessary forces to move the teeth are light and continuous [20] which would tend to physiologically preserve the periodontal ligament [44]. The force magnitude did not appear to be decisive for the incidence of root resorption. This means that light forces can cause extensive root resorption too [45]. Other studies have also shown no differences between conventional and self-ligating brackets [21, 22, 41, 46, 47]. Leite et al. [22] demonstrated that root resorption is similar in self-ligating and conventional ligating brackets during the initial treatment stage with CBCT that provides greater image accuracy. Future comparative studies with CBCT after complete treatment should be performed to confirm the current results.

Despite there were no intergroup significant differences in the degree of root resorption, group 2 (preadjusted) had more patients with score 0 when compared to group 1. Group 1 (Damon) showed more patients with score 1 than group 2. Regarding moderate resorption, group 2 had more patients than group 1. Accentuated resorption was present in both groups similarly (Table 3).

Regardless of the technique (preadjusted or self-ligating appliances), low rate of EARR was expected because the sample consisted of patients with only moderate crowding and all were treated non-extraction. The results showed that 49.7 % of the teeth had no root involvement. Grade 3 (accentuated resorption) was present in only 1.9 % of the cases, while extreme resorption was not present (Table 4). These results are similar to the other studies [29, 48]. However, these results differ from those of DeShields [7] who found resorption in 99.08 % of patients. Probably this was because only the maxillary incisors were evaluated and resorption was assessed on the lateral headfilms, which prevents correct visualization of the apices due to overlapping of anatomical structures.

Although there were no statistically significant differences regarding the amount of resorption in conventional and passive self-ligating systems, some mechanical considerations are in order.

Low resistance to sliding is one of the most important advantages attributed to self-ligating appliances. The light force released by this system probably is its main feature that would make them better than conventional. It is claimed that self-ligating brackets, besides requiring lighter forces for dental movement, due to reduced resistance, are also able to preserve it longer because of the wire/bracket attachment. However, this factor did not have an effect on the amount of root resorption in both groups.

A sequence of round section and low gauge NiTi archwires were employed in both groups in this research during the initial stage (leveling and alignment). A small variation in the intermediate stages of treatment in archwire sequence was due to the mechanotherapy scheme suggested by Damon. Conventional Roth and standard Damon have the same torque and same angulation on the incisors, suggesting that these factors did not affect the amount of resorption. Similarly, the tying method in the conventional brackets, by means of elastomeric ligatures, as compared to the self-ligating brackets, did not seem to have influenced the amount of root resorption in both groups. Similarly, a systematic review concluded that archwire sequence, bracket prescription, and tying method did not affect the amount of external apical root resorption and that heavy force application was one of the most important factors for severe resorption [16].

Independent of force magnitude and force regimen, large individual variations in EARR levels are expected, considering the individual metabolic response to mechanical stimuli. This means that the cause of EARR is multifactorial and that some individuals are predisposing to EARR, but at the moment, it is still not possible to identify these patients at risk before the start of orthodontic treatment [45, 49].

The similarity in root resorption in both groups cannot be used as a selection criterion between them. The continuous development and evolution of orthodontic materials have been providing greater comfort for the professional, characterized by decreased chair time and need to exchange the wires. Patients have also been favored, because the new materials quickly improve the smile esthetics, due to the early leveling and alignment provided by reduced friction between wire and bracket. It is upon the clinician to select the most appropriate appliance, considering its advantages, disadvantages, and treatment time.

## Conclusions

There was no significant difference in the external apical root resorption between patients treated with the Damon self-ligating system or with conventional ligating appliances.
